# Proceedings of Societies

**Published:** 1871-09

**Authors:** 


### Proceedings of Societies.

### ILLINOIS STATE MEDICAL SOCIETY.

The Annual Meeting of this Society was held in Peoria, commencing on Tuesday morning, May 16th, and continuing until one o'clock P. M. of the Thursday following. It was one of the largest and best meetings that have been held since the organization of the Society in 1850. The following record embraces the most important part of the proceedings:

The Society was called to order at ten o'clock, by the President, Dr. G. W. Albin.

The roll of officers was called and all found to be present, as follows:

*President*—G. W. Albin, M.D., of Neoga.

*First Vice President*—John Murphy, M.D., of Peoria.

*Second Vice President*—J. S. Whitmire, M.D., of Metamora.

*Treasurer*—J. H. Hollister, M.D., of Chicago.

*Permanent Secretary*—T. D. Fitch, M.D., of Chicago.

*Assistant Secretary*—J. P. Johnson, M.D., of Peoria.

*Committee of Arrangements*—Drs. J. C. Frye, R. Boal, J. O. Hamilton, R. Roskoten, Peoria; S. Maus, Pekin; J. S. Whitmire, Metamora.

Drs. Peck and Baxter, delegates from the Iowa State Medical Society, were introduced by Dr. Plummer, of Rock Island, and were invited to take seats and participate in the business of the meeting as delegates. Dr. Peck responded briefly, and invited the Society to appoint delegates to attend the next annual meeting of the Iowa Society.

On motion of Dr. J O. Hamilton, the annual assessment for each member was fixed at $3.00.

The hearing of reports from standing committees being in order, Dr. H. A. Johnson, of Chicago, chairman of the Committee on Practical Medicine, gave a verbal abstract of a somewhat lengthy and interesting report, the materials for which had been obtained chiefly from the records of the Chicago Board of Health.

At the close of the report a recess was taken for dinner.

AFTERNOON SESSION.

The Society was called to order at two o'clock P.M., President Albin in the chair.

Dr. Boal, in behalf of the Committee of Arrangements, delivered a brief and very appropriate address of welcome; and several additional members presented their credentials and were registered.

The consideration of the report of the Committee on Practical Medicine was resumed, and Dr. G. B. Hawley, of Aurora, inquired if the report contained anything in regard to the condition of certain classes of people in different parts of the city, the actual hygrometric condition of the atmosphere, as well as the amount of rain fall, etc.

Dr. H. A. Johnson stated that the report did contain details in regard to all the matters mentioned by Dr. Hawley, except that of the hygrometric condition of the atmosphere, and he stated some striking facts illustrating the effects of sewerage and pure water in lessening the mortality. He also stated that the south and south-east winds were always followed by an increase in the daily number of deaths in the city.

Dr. Hewett stated that in his locality in Jersey county he had observed an aggravation of disease pretty uniformly in connection with dry south and south-east winds. The subjects embraced in the report were further discussed by Drs. D. W. Young, of Aurora, J. H. Hollister, and N. S. Davis, of Chicago. The latter stated that temperature and moisture certainly exert a great influence in the production of diseases, but in the production of bowel affections, so prevalent, especially among children in the summer, another element acted in conjunction with the heat and moisture. That element was some kind of atmospheric impurity. He had observed that the bowel complaints of children in Chicago invariably commence with the first week of continuous hot weather after the fresh fallen surface water of spring has disappeared, so that the surface soil is simply moist, or in a process of becoming dry. It is then that the processes of organic change and decompositi on are most rapid, and the products impregnate the atmosphere to the greatest extent.

Dr. J. C. Corbus, of Mendota, member of the Committee on Practical Medicine, made a supplementary report in relation to the diseases of his locality, which, together with the report of Dr. Johnson, was referred to the Committee of Publication.

The President then announced that the Mayor and City Attorney of Peoria were present, and desired an audience with the Society.

The Mayor introduced Mr. Quinn, the City Attorney, who cordially welcomed the Society to the city.

Dr. H. A. Johnson responded on behalf of the Society.

Dr. Edmund Andrews, of Chicago, chairman of the Committee on Surgery, presented an interesting report on various surgical topics, embracing improvements in the operation for phymosis by Prof. J. S. Sherman, of Chicago; improved instruments for operating for cleft palate, irrigating the urethra, suppressing hemorrhage, using the endoscope, the choice of anæsthetics, the effects of transplantation of skin and cuticle in the healing of chronic ulcers, etc. He related three cases of the latter process by himself, and alluded to one by Prof. E. Powell.

Dr D. Prince, of Jacksonville, stated that he had made several experiments in the transplantation of cuticle to the surface of chronic ulcers. In all of his cases the ulcers were suppurative, and the experiments failed. He had been informed by Dr. Hodgen, of St. Louis, that his trials in ulcers of a similar kind had also failed, but when the transplantation had been to the surface of ulcers that were dry or non-suppurative, it had been followed by a satisfactory degree of success.

Dr. Andrews, in his report, had recommended a row of gas jets ten inches long for illuminating the urethra and other internal parts, and Dr. H. A. Johnson inquired whether the color of the light needed any correction in order to appreciate correctly the color of the illuminated surfaces?

Dr. Andrews replied that the light was sufficiently accurate for practical purposes.

Dr. Johnson added that he had found an ordinary Argand burner with a deep blue chimney to give the color of tissue very perfect. He also stated that recent cases of gonorrhœa could be generally rendered abortive, or speedily cured, by the local application of a solution of permanganate of potassa, 10 grs. to the oz. of water. The application is unirritating and painless.

Dr. D. Prince said that tannic acid, moistened with glycerine and made into the form of a bougie. when introduced into the urethra and left to dissolve in the moisture of the mucous membrane, generally rendered recent gonorrhœa abortive.

Dr. Kilbourn spoke against the use of ordinary astringents in the treatment of this disease, and recommended the method of irrigation described in the report of the committee.

Dr. T. D. Fitch thought the method of operating for phymosis adopted by Dr. J. S Sherman, as described in the report, was not very different from that recommended by Erichsen, in his work on surgery, and which he had followed in his own practice with entirely satisfactory results. In regard to the use of anæsthetics in surgery, he stated that up to the last two years he had given the preference to chloroform, and had so expressed himself in the anniversary meeting of this Society for 1869. But since that time his personal observations, as well as the accumulated facts in our medical literature, had induced a change in his mind, and he now thought chloroform should be used only in exceptional cases. He still entertained fears that though ether was much less dangerous in its immediate effects, yet its secondary influence on the blood might materially retard or aid in preventing the final recovery of the patient.

Dr. Harriot inquired how Drs. Fitch and Andrews administered chloroform, and was informed that they used it on a napkin over the mouth and nostrils of the patient, holding it in such a position as to allow a reasonable admixture of atmospheric air. He stated that he had used chloroform very often for a number of years without any bad results.

Dr. J. H. Hollister inquired whether any one had known fatal results from the inhalation of chloroform after the preceding inhalation of ether?

Dr. Mosher said that Dr. Frank H. Hamilton, of New York, gave ether to a patient, and failing to produce the desired anæsthesia, followed it by chloroform. which proved speedily fatal.

Dr Traverse had given chloroform frequently in obstetric practice without any dangerous effects, but thought there was more danger from hemorrhage after the delivery; so much so, that he now pretty generally gave a dose of ergot immediately after the completion of the labor.

Dr. White, of Bloomington, also thought that the use of chloroform increased the tendency to excessive flowing after labor. He related a death from the inhalation of chloroform used by mistake, supposing it to be ether.

The *post mortem* showed the blood to be in a fluid condition, and a specimen of it kept in a vial several months presented no coagulation.

Dr. H. A. Johnson alluded to a fatal case of poisoning by hydrate of chloral, recently published in Europe, in which the *post mortem* revealed the presence of chloroform in the blood, and made some comments on the philosophy of anæsthesia.

Dr. Crawford said he had used chloroform as the principal anæsthetic in his practice, using it often, and without any serious results. He thought much of the safety or danger incurred in its use depended on the mode of its administration. The method which he learned while connected with the army was to lay one or two thicknesses of factory cloth over the mouth and nostrils of the patient and then put the chloroform on it drop by drop.

Dr. J. S. Whitmire practiced and recommended the same method, and though he had given the chloroform a great many times, and still adhered to its use, he had met with no bad results. He believed, however, that its use should be limited to really important cases of surgery, and strongly condemned its use for extracting teeth and other trivial operations.

Dr. S. J. Jones and Dr. W. Young coincided with Drs. Crawford and Whitmire in relation to the method of using chloroform. The latter gentleman stated that the reason why so few accidents had resulted from the use of chloroform in obstetrics compared with general surgery was, because in labor it is given to allay pain already present, while in surgery it is given in advance, to prevent suffering anticipated.

Dr. Jenks stated that he had found a mixture of chloroform, tinct. opii., and tinct. aconite, applied locally to the gums, to produce all the anæsthesia necessary to extracting teeth.

On motion, the report of Dr. Andrews on surgery was referred to the Committee of Publication.

The Society then adjourned until 8 o'clock P. M.

EVENING SESSION.

On re-assembling at eight o'clock in the evening, Dr. Albin in the chair, Dr. J. Murphy, of Peoria, made an interesting verbal report concerning the method of treating strictures of the urethra. For all old and indurated strictures he recommended the use of chromic acid, applied directly to the stricture. He introduces a large-sized bougie, with several holes in the end, presses it down upon the stricture and injects through it the solution of chromic acid, repeating the application at first twice a day. When the tissue of the stricture has been softened by the application, a small-sized bougie, with holes in the sides, is used and pressed through the stricture, and acid injected as before. He continues this once a day, with increased size of the instrument, until a No. 8 passes readily. He commences the use of a solution of chromic acid, 40 grs., water, ℥j, but increases the strength gradually to 60 grs. to the ounce of water.

Dr. D. Prince, of Jacksonville, member of the Committee on Surgery, made an interesting and lengthy report on the improvements in the departments of plastic and orthopœdic surgery, which was referred to the Committee on Publication.

The Committee on Obstetrics and Diseases of Women, Dr. DeLaskie Miller, of Chicago, chairman, and the Committee on Criminal Abortion, Dr. W. H. Byford, of Chicago, chairman, were called, but no reports were made.

The Committee on Ophthalmology, Dr. H. H. Roman, of Springfield, chairman, was called, but no report from the chairman. Dr. E. L. Holmes, of Chicago, one of the committee, read a short report on cataract, which was referred to the Committee of Publication.

The Society then adjourned until eight o'clock the next morning.

WEDNESDAY MORNING.—SECOND DAY.

The Society was called to order at eight o'clock A.M., Dr. G. W. Albin in the chair.

Dr. Boal, chairman of the Committee of Arrangements, reported that the committee had provided a new meeting place in the basement of the Universalist Church.

Dr. Davis moved that the meeting adjourn to the new rooms selected, but withdrew his motion, as it was desirable to remain in the hall to witness the exhibition of Dr. Truesdale's fracture-bed, after which the Society adjourned to the lecture-room of the Universalist Church. The new place of meeting is much more suitable than Rouse's Hall was. The acoustic qualities of the room are better; it is not so large, and therefore better suited to the use of the 100 or 150 delegates, and the seats and general appointments are much more comfortable. The Society were compelled to change their place of meeting on account of a minstrel performance.

The following names were proposed for permament membership: Drs. J. P. Walker and N. Elsbury, of Mason City, Ill., by Drs. D. Prince and E. Andrews; Dr. C. D. Knapp, of San Jose, and Dr. N. B. Cole, of Bloomington, by Drs. R. D. Bradley and J. S. White; and Dr. John Gregory, by Drs. J. W. Hensley and H. Steele.

On suggestion of Dr. Davis, a recess of ten minutes was taken to receive the names of the nominating committee. When the meeting was called to order, the Committee on Nominations reported.

Dr. David Prince, of Jacksonville, then introduced to the Society Drs. Wm. M. McPheeters and Wm. S. Edgar, of St. Louis, and moved that they be allowed the rights and privileges of delegates, which motion was unanimously adopted.

Dr. Johnson, of Peoria, continued the report on ophthalmology, in the case of ocular hyperæsthesia, which was discussed by Drs. Warner, McFarland, Johnson, Littlefield, and Holmes, and on motion, the matter was referred to the Committee of Publication.

The Committee on Phthisis Pulmonalis was called, when it was announced by the President, that the chairman, Dr. Noble, was dead.

The Committee on Otology was called, and the chairman, Dr. S. J. Jones, of Chicago, made his report, which was discussed by Dr. E. L. Holmes.

On motion, the report of the Committee on Otology was accepted, and referred to the Committee of Publication.

The Committee of Investigation reported favorably on a number of names presented as permanent members, and they were declared elected.

The Committee on Nominations made the following partial report:

Dr. J. O. Hamilton, of Jerseyville, President; Dr. T. Worrel, of Bloomington, First Vice-president; Dr. D. W. Young, of Aurora, Second Vice-president; Dr. J. H. Hollister, of Chicago, Treasurer.

On motion, this report was adopted, and the candidates elected unanimously; and on further report of the committee, Rock Island was selected as the next place of meeting.

The Committee on Criminal Insanity being called, Dr. McFarland, the chairman, submitted a verbal report, which was discussed by Drs. McCartney, of Young America, and N. S. Davis, of Chicago.

The Committee on Idiocy was called, and Dr. C. T. Wilbur, of Jacksonville, presented his report, which on motion was adopted, and referred to the Committee of Publication.

On motion, the Society then adjourned.

In the afternoon, an excursion by rail was taken to Prospect Hill. The train stopped at the residence of R. M. Cole, Esq., where three fine photographs were taken of the Society.

In the evening, a reception and banquet was held at Parmely's Hall.

THURSDAY MORNING.—THIRD DAY.

The Society was called to order, at 9 A. M.; President Albin in the chair. The reading of the minutes was dispensed with, and the regular order of reports of committees proceeded with.

Dr. N. S. Davis, chairman of a special committee, read a report on the internal use of carbolic acid in the treatment of diseases, which was referred to the Committee of Publication.

Dr. D. W. Young, Special Committee on Cholera Infantum, read a lengthy and interesting report on that subject, which was referred to the Committee of Publication.

Dr. T. D. Fitch, Special Committee on Bowel Affections of Children, gave a verbal statement of his report on the nature and treatment of constipation in infants. The statement was accepted, and the author requested to furnish his report to the Committee of Publication. All these reports were listened to with much interest, and would have elicited very profitable discussion had not the hour for final adjournment been so near at hand.

Dr. J. S. Whitmire, Special Committee on the Duty of the Profession towards the State Medical Society, read a report, which was accepted, and referred to the Committee of Publication.

A paper on Criminal Abortion, by Dr. A. Niles, of Quincy, had been received through the Special Committee on that subject. The author not being present to read his paper, it was referred to the Committee of Publication, with instructions to examine the same and publish or not as they thought best.

The annual reports of the Treasurer and of the Publication Committee were presented, accepted, and placed on file.

They showed the financial condition of the Society highly satisfactory and prosperous.

Delegates were then elected to represent the Society in the next annual meeting of the American Medical Association, as follows:

Dr. N. S. Davis, Chicago; Dr. F. B. Haller, Vandalia; Dr. J. P. Johnson, Peoria; Dr. M. W. Walton, Ridott; Dr. J. O. Hamilton, Jerseyville; Dr. D. M. Vosburgh, Earlville; Dr. Geo. J. Monroe, Leland; Dr. D.W. Young, Aurora; Dr. E. P. Cook, Mendota; Dr. J. L. White, Bloomington; Dr. E. Willard, Wilmington ; Dr. C. Winney, Sandwich ; Dr. John Murphy, Peoria; Dr. D. W. Jones, Danville; Dr. David Prince, Jacksonville; Dr. J. H. Hollister, Chicago; Dr. C. Truesdale, Rock Island; Dr. H A. Johnson, Chicago; Dr. E. Andrews, Chicago; Dr. D. E. Foote, Belvidere; Dr. T. D. Fitch, Chicago; Dr. S. P. Hawley, Aurora; Dr. N. G. Blalock, Mount Zion; Dr. D. S. Jenks, Plano; Dr. Lucius Clark, Rockford; Dr. W. M. Kaul, Limerick; Dr. E. M. Travers, Amboy; Dr. Geo. W. Wright, Canton; Dr. C. Goodbrake, Clinton; Dr. Andrew McFarland, Jacksonville.

Delegates were also elected to the State Medical Societies of the adjoining States of Iowa, Missouri, and Indiana, as follows: To Missouri, Drs. G. W. Hall, of Carthage; F. B. Haller, of Vandalia; J. O. Hamilton, Jerseyville; and T. J Pitman. To Iowa, Dr. Samuel C. Plummer, of Rock Island; and to Indiana, Dr. Wm. H. Byford, of Chicago.

A number of resolutions were adopted tendering thanks to the Committee of Arrangements, the proprietors of the hall and church in which the meetings were held, to the railroads, to the city authorities and citizens generally of Peoria for the liberal and excellent arrangements made and executed for the accommodation of the Society during the meeting about to close. The thanks of the Society were also tendered to the retiring President, for the able and faithful manner in which he had discharged his official duties.

The President, Dr. G. W. Albin, responded briefly, and the Society adjourned, to meet in Rock Island on the third Tuesday in May, 1872.—Abridged from *Exchange*, (in the absence of the Secretary).

---

### *Illinois Institution for the Education of Feeble Minded Children.*

This Institution, which was inaugurated in 1865 as an experimental school for the education of feeble minded children, has been so successful in training this unfortunate class that at the last session of the General Assembly it was organized upon an independent basis, and was incorporated as one of the permanent charitable institutions of the State, thus completing the noble circle of public charities of the commonwealth of Illinois.

The design and object of the Institution is to furnish the means of education to children and youth of feeble minds, who are deprived of educational privileges elsewhere, and who are of a proper school-attending age. It is designed for those so deficient in intelligence as to be incapable of being educated at common schools, who are not epileptic, insane or deformed.

The education furnished by the Institution will include, not only the simple elements of instruction usually taught in common schools, where that is practicable, but will embrace a course of training in the more practical matters of every-day life; the cultivation of habits of decency, propriety, self-reliance, and the development and enlargement of a capacity for useful occupation.

The combination which this Institution presents, of practical medical care and proper physical and mental training, with efficient educational resources, will supply, it is hoped, a want which has long been felt and imperatively demanded by this unfortunate class of children and youth of the State.

The improvement and progress of the pupils have been very encouraging, and parents and friends in almost every instance have expressed satisfaction with what has been accomplished in the short time since the school was organized.

The Institution is open to the inspection of the public at all reasonable hours; and all are not only cordially invited, but are earnestly requested to visit the school.

It is a State Institution, and board and tuition are free during the school year of ten months.

It is the desire of the Trustees to ascertain accurately the number of this unfortunate class of persons in the State, and persons knowing the residence of any in Illinois will confer a favor by reporting the same to the undersigned, as it is desirable that reliable statistics may be gathered in order that proper legislation may be made in their behalf.

Those designing to apply for the admission of pupils should do so at once as the accommodations are limited.

Applications for admission, information, etc., should be directed to

Dr. C. T. Wilbur, *Superintendent*,
Jacksonville, Illinois.

